# A prognostic model for oral squamous cell carcinoma using 7 genes related to tumor mutational burden

**DOI:** 10.1186/s12903-022-02193-3

**Published:** 2022-04-29

**Authors:** Fei Wu, Yuanyuan Du, Xiujuan Hou, Wei Cheng

**Affiliations:** 1 Department I of Oral Comprehensive Outpatient, Yantai Stomatological Hospital of Binzhou Medical University, Yantai, 264001 Shandong China; 2 Department of Dental Implant, Yantai Stomatological Hospital of Binzhou Medical University, Yantai, 264001 Shandong China; 3 Department of Dental Prosthodontics, Yantai Stomatological Hospital of Binzhou Medical University, No. 142 Zhifu District, Yantai, 264001 Shandong China

**Keywords:** Oral squamous cell carcinoma (OSCC), Tumor mutational burden (TMB), Differential expression genes (DEGs), Risk Score

## Abstract

**Background:**

Oral squamous cell carcinoma (OSCC) is a rising problem in global public health. The traditional physical and imageological examinations are invasive and radioactive. There is a need for less harmful new biomarkers. Tumor mutational burden (TMB) is a novel prognostic biomarker for various cancers. We intended to explore the relationship between TMB-related genes and the prognosis of OSCC and to construct a prognostic model.

**Methods:**

TMB-related differential expressed genes (DEGs) were screened by differential analysis and optimized via the univariate Cox and LASSO Cox analyses. Risk Score model was constructed by expression values of screened genes multiplying coefficient of LASSO Cox.

**Results:**

Seven TMB-related DEGs (CTSG, COL6A5, GRIA3, CCL21, ZNF662, TDRD5 and GSDMB) were screened. Patients in high-risk group (Risk Score >  − 0.684511507) had worse prognosis compared to the low-risk group (Risk Score <  − 0.684511507). Survival rates of patients in the high-risk group were lower in the gender, age and degrees of differentiation subgroups compared to the low-risk group.

**Conclusions:**

The Risk Score model constructed by 7 TMB-related genes may be a reliable biomarker for predicting the prognosis of OSCC patients.

**Supplementary Information:**

The online version contains supplementary material available at 10.1186/s12903-022-02193-3.

## Background

Oral cancer is the sixth most common cancer type in the world [1]. Oral squamous cell carcinoma (OSCC) is the most commonly occurring oral cancer [2]. The OSCC represents a major public health issue, especially in the developing countries for example China [3]. OSCC usually arises from and develops in the oral cavity and oropharynx [4], which can induce damage in speech, swallowing and chewing function [2]. The risk factors of OSCC include smoking, excessive alcohol consumption, areca nut chewing (especially in Asia and Pacific area), occupational exposure to carcinogens, autoimmune chronic disease, persistent viral infections (e.g. human papillomavirus, HPV) and so on [5, 6]. Treatment options for the OSCC patients comprise of surgical resection, adjuvant radiotherapy and chemotherapy, as well as the rising immunotherapy [7–9]. But due to the tendency to metastasize [10], patients with advanced OSCC are likely to have a poor prognosis [11]. Traditional prognostic indicators for example stages and grades of tumor are difficult to distinguish carcinomas with different biological characteristics within the same histological subgroup [12, 13]. Novel indicators such as immune-related genes [14], systemic inflammatory biomarkers [15], ferroptosis-related genes [16] are emerging as effective biomarkers to stratify patients with different prognosis. These identified biomarkers provide a relatively comprehensive understanding of prognosis in OSCC and provide an additional tool for selecting patients who need more aggressive treatment. In order to improve accuracy of the prediction, more biomarkers are urgently needed to be explored to provide an additional tool for prediction of prognosis for cancer patients [17, 18].

Tumor mutational burden (TMB) is defined as the number of mutations existing within a tumor and is often reported as the number of mutations per DNA megabase of genomic territory [19]. Because of the development of next generation sequencing techniques, a cost- and time-effective sequencing of genes makes significant improvement in detecting gene mutations [20]. Growth and progression of cancers are reported to be related to the immune suppression, and in order to evade immunosurveillance and eradication of the host immune system, tumors often upregulate immune checkpoints [21, 22]. Immunotherapies based on immune checkpoint inhibitors (ICIs) have emerged as a new treatment for many types of cancers [23]. High TMB levels is often connected with better prognosis and higher rates of treatment response after ICIs therapy which may attribute to higher potential immunogenic neoantigens facilitating anti-tumor immune response [24, 25]. And TMB levels are emerging as a novel prognostic biomarker for the response to immunotherapy in oncology clinic [21, 26, 27]. Previous studies have reported that cancer patients with higher TMB levels have higher response rates following ICI therapy than those with lower TMB levels, for example non-small cell lung cancer (NSCLC) [28], melanoma [29] and breast cancer [26]. TMB levels are used for the prediction of the prognosis for cancer patients following immunotherapy in solid tumors such as breast cancer, lung cancer and gastrointestinal cancers [30]. And Kang et.al. have reported that TMB was also related to the prognosis of cutaneous melanoma and prognostic model constructed by TMB-related grenes might be used to predict prognosis of cancer patients [31]. These researches support that TMB has the potential as a promising biomarker for predicting the cancer patients with different prognosis [32]. Although previous studied have identified the prognostic signature constructed by TMB-related genes for patients with ovarian cancers [33] and the prognostic value of TMB for patients with head and neck squamous cell carcinoma has also been studied [34], there are few articles about the prognostic value of TMB-related genes for OSCC patients and the prognostic signature constructed by TMB-related genes for OSCC patients has not been throughly explored. We aimed to explore the prognostic value of TMB-related genes for and to build a prognostic signature for OSCC patients. Besides, Risk Score models constructed by molecular biomarkers utilizing LASSO Cox regression analysis have already been used to diagnose and to predict the prognosis of patents in solid tumors [35].

In this study, we explored the connection between TMB-related genes and the prognosis of OSCC patients through bioinformatic analysis. We hoped to construct a prognostic Risk Score model to be helpful in separating patients with different prognosis.

## Material and methods

### Data collection

Data of the mRNA expression and clinical information about 306 OSCC patients were downloaded from the Cancer Genome Atlas (TCGA, https://tcga-data.nci.nih.gov/tcga/) database. The full clinical information of 306 OSCC patients was shown in Table 1. Maf files of 311 OSCC patients were also downloaded from the TCGA database for further analysis and clinical information of 311 OSCC patients was listed in Table 2. Moreover, the dataset GSE41613 was downloaded from the Gene Expression Omnibus (GEO, https://www.ncbi.nlm.nih.gov/geo/) database. The dataset was comprised of 97 OSCC patients with complete survival information. The GEO dataset was derived from HPV-negative OSCC patients, while the HPV status of TCGA cohort was unknown. Patients’ data were processed by the Affymetrix Human Genome U133 Plus 2.0 Array.

**Table 1:** Clinicopathological characteristics of OSCC patients from TCGA database

Characteristics	Patients (N = 306)	
	No	%
Gender		
Female	102	33.33
Male	204	66.67
Age		
≤ 61 (Median)	157	51.31
> 61 (Median)	149	48.69
Grade		
GX	3	0.98
G1	49	16.01
G2	191	62.42
G3	62	20.26
Unknown	1	0.33
Survival time		
Long (> 5 years)	31	10.13
Short (< 5 years)	275	89.87
OS status		
Dead	143	46.73
Alive	163	53.27
M		
M0	289	94.44
M1	2	0.65
Mx	12	3.92
Unknown	3	0.98
N		
N0	160	52.29
N1	56	18.30
N2	76	24.84
N3	2	0.65
Nx	9	2.94
Unknown	3	0.98
T		
T1	18	5.88
T2	97	31.70
T3	73	23.86
T4	110	35.95
Tx	5	1.63
Unknown	3	0.98
Primary site		
Anterior floor of mouth	2	0.65
Border of tongue	1	0.33
Cheek mucosa	19	6.21
Floor of mouth	51	16.67
Gum	8	2.61
Hard palate	4	1.31
Lip	3	0.98
Lower gum	2	0.65
Mouth	20	6.54
Overlapping lesion of lip, oral cavity and pharynx	69	22.55
Palate	1	0.33
Tongue	125	40.85
Upper gum	1	0.33

**Table 2:** Clinicopathological characteristics of OSCC patients from TCGA database

Characteristics	Patients (N = 311)	
	No	%
Gender		
Female	107	34.41
Male	204	65.59
Age		
≤ 61 (Median)	159	51.13
> 61 (Median)	152	48.87
Grade		
GX	3	0.96
G1	50	16.08
G2	196	63.02
G3	61	19.61
Unknown	1	0.32
Survival time		
Long (> 5 years)	32	10.29
Short (< 5 years)	279	89.71
OS status		
Dead	146	46.95
Alive	165	53.05
M		
M0	294	94.53
M1	2	0.64
Mx	12	3.86
Unknown	3	0.96
N		
N0	162	52.09
N1	57	18.33
N2	78	25.08
N3	2	0.64
Nx	9	2.89
Unknown	3	0.96
T		
T1	18	5.79
T2	98	31.51
T3	77	24.76
T4	110	35.37
Tx	5	1.61
Unknown	3	0.96
Primary site		
Anterior floor of mouth	2	0.64
Border of tongue	1	0.32
Cheek mucosa	19	6.11
Floor of mouth	53	17.04
Gum	8	2.57
Hard palate	4	1.29
Lip	3	0.96
Lower gum	2	0.64
Mouth	21	6.75
Overlapping lesion of lip, oral cavity and pharynx	67	21.54
Palate	1	0.32
Tongue	129	41.48
Upper gum	1	0.32

### Differential analysis

Analysis of differential expression genes (DEGs) was based on limma function package [36] of R programming software (version4.1.0, the same below). The |Log_2_FC|> 1 and adjusted *P* value ≤ 0.01 were used to screen DEGs associated with TMB.

### Functional enrichment analysis

Functional enrichment analysis was applied to the TMB-related DEGs using the “clusterProfiler” package [37] of R programming software. The Kyoto Encyclopedia of Genes and Genomes (KEGG) pathways and Gene Ontology (GO, including Biological Process, Molecular Function and Cellular Component) terms were used to examine the enriched GO terms and KEGG pathways. Threshold of adjusted *P* value < 0.05 of Benjamini and Hochberg (BH) method were used to screen significantly enriched GO terms and KEGG pathways.

### Construction of prognostic Risk Score model

Univariate Cox regression analysis, based on the expression values of the 324 TMB-related DEGs, was performed on the 306 OSCC patients with the threshold of *P* < 0.01 to screen the DEGs which were associated with prognosis of OSCC. LASSO Cox regression analysis was performed on the screened DEGs to optimize the genes using the glmnet package [38] of R programming software. Then Risk Score of each patient was calculated using the following formula based on the screened TMB-related DEGs:

$${\text{Risk}}\;{\text{score}} = \sum\limits_{{{\text{i}} = 1}}^{{\text{n}}} {{\text{Coef}}_{{\text{i}}} *{\text{X}}_{{\text{i}}} ,}$$

In this formula, Coef_i_ and X_i_ are the coefficient calculated by LASSO Cox and expression value of each gene (the expression value of mRNA in this research) respectively. Survival, survminer and two-sided log-rank test of R package were used to test the Risk Scores. Patients were assigned into low-risk and high-risk groups according to the median of Risk Score.

### Survival analysis

We used the survival and survminer packages of R programming software to estimate the OS rates of different groups using Kaplan–Meier method. The significance of difference of OS rates between different groups was tested by log-rank test. The independence of Risk Score in predicting the prognosis of OSCC patients was examined by multivariate Cox regression analysis. Age and degree of differentiation were included in the study as the clinicopathological factors affecting the prognosis of many cancers. The TNM status was included in the multivariate Cox regression analysis as the reliable maker for treatment decision but the tumor size or/and extension (T) was excluded because of its inadaptability to the regression model. The four factors and Risk Score were included in the multivariate Cox regression analysis as variables.

### Construction of nomogram model

Nomogram is often used to predict the prognosis of many types of cancers. In our study, nomogram constructed by the independent factors was built to predict 1-year, 3-year and 5-year OSs of patients. We used rms (https://CRAN.R-project.org/package=rms) package of R programming software to build nomogram. In order to observe the accuracy of the predicted probability, a calibration curve was drawn.

### Calculation of immune cell infiltration proportion

CIBERSORT algorithm [39] was based on gene expression matrix. It used predisposed 547 barcode genes and employed deconvolution method to characterize the composition of immune infiltration cells. And CIBERSORT was used to calculate the infiltration proportion of 22 immune cells of each patient. The sum of estimated proportions of all immune cells in each patient was 1.

## Results

### Identification of DEGs

**Fig. 1:**
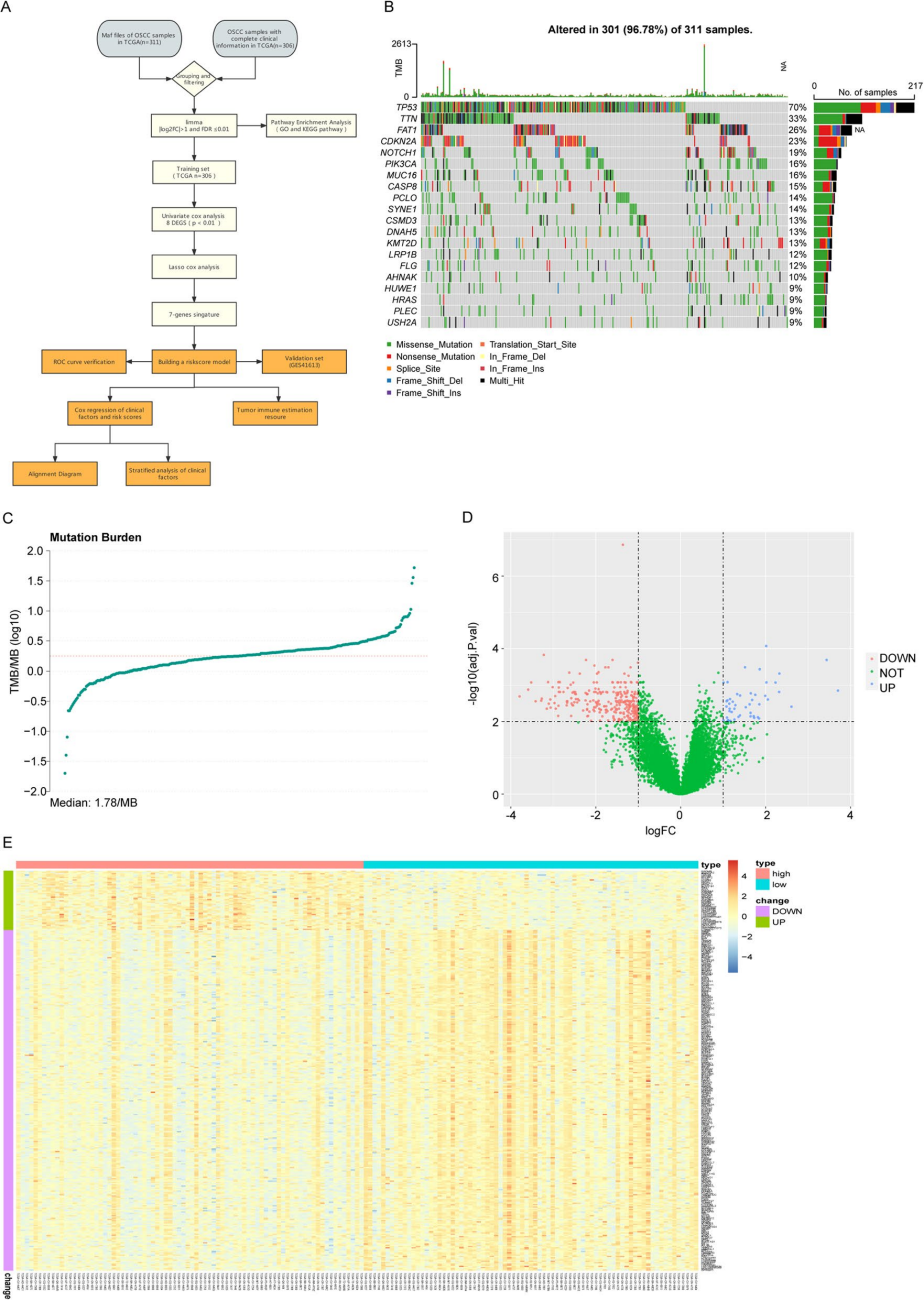
Mutation status and differential gene analysis of OSCC in TCGA. **A** Flow diagram of the study. **B** Waterfall diagram of the top 20 genes with the highest mutation rate of OSCC in TCGA. **C** The distribution graph of TMB value. The horizontal coordinate is TMB value and the vertical coordinate is TMB value log base 10. **D** The volcano diagram of TMB-related genes. The horizontal axis is differentially expressed multiple (Log_2_FC) and the vertical axis is − log10 (adjusted *P* value). The blue dots and the red dots represent up-regulation genes and down-regulation genes. **E** Heatmap of TMB-related genes, the horizontal axis and the vertical axis are the patients and different genes. Red and blue represent high expression and low expression of genes and green and purple represent up-regulated and down-regulated genes

Figure 1A was a flow diagram of study to clarify the design of our study. We processed the maf files of 311 OSCC patients using maftools package of R programming software and the results showed TP53, TTN and FAT1 had the highest mutation rates (Fig. 1B). 161 patients with TMB values in the front 25% (≤ 1.16) and the back 25% (≥ 2.38) were divided into the low-TMB group and high-TMB group (Fig. 1C). Then 157 mRNA expression profiles were found from the 306 patients. 324 DEGs associated with TMB were screened out of the 157 patients, using the limma function package of R programming software. In the high- vs low-TMB group, up-regulated genes and down-regulated genes were 48 and 276 respectively (Fig. 1D). Expression levels of DEGs were significantly different between high- and low-TMB groups (Fig. 1E).

### Go terms and KEGG pathway analysis of DEGs

**Fig. 2:**
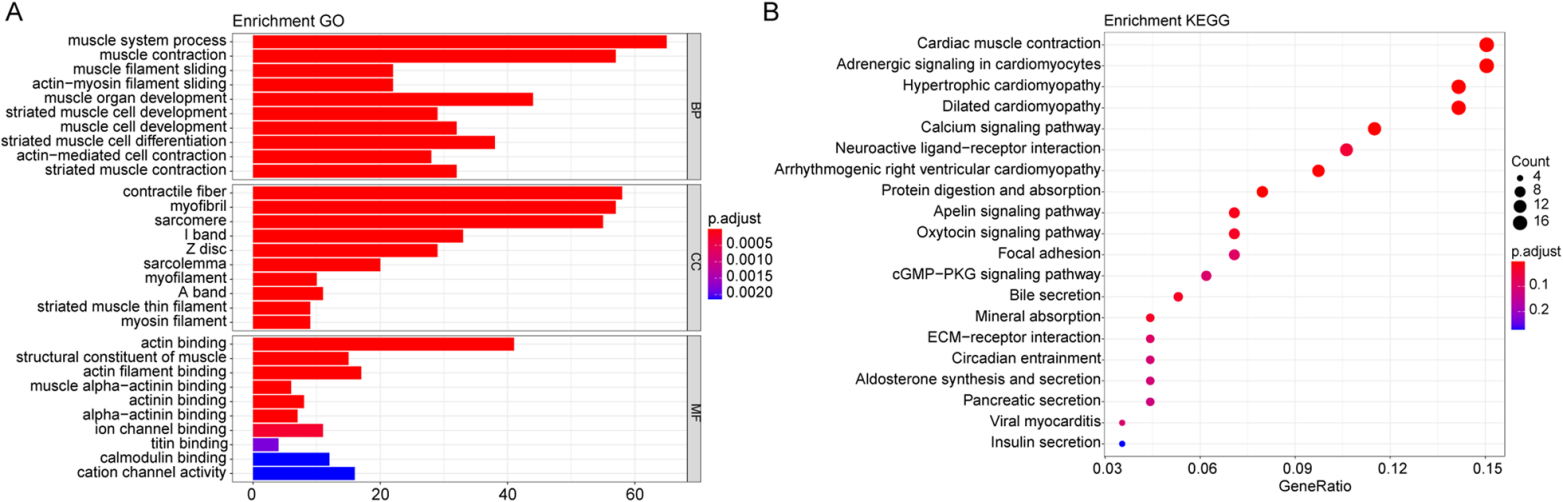
Functional enrichment analysis results. **A** GO enrichment analysis of TMB-related DEGs of, including biological process (BP), cellular component (CC) and molecular function (MF). The x axis is the number of genes and the y axis is the GO terms. **B** Functional enrichment analysis of TMB-related DEGs in KEGG Pathways. The x axis is the number of genes and the y axis is the KEGG pathways

GO and KEGG enrichment analyses were done to the 324 TMB-related DEGs. Significant enrichment terms were found among the muscle system process, contractile fiber, actin binding in GO terms and Cardiac muscle contraction in KEGG Pathways. Top 10 Go terms and top 20 KEGG pathways of the TMB-related genes were shown on Fig. 2A, B. Full results of Go and KEGG enrichment analyses were listed on Additional file 1: Table S1 and Additional file 2: Table S2.

### Construction and validation of Risk Score model

**Fig. 3:**
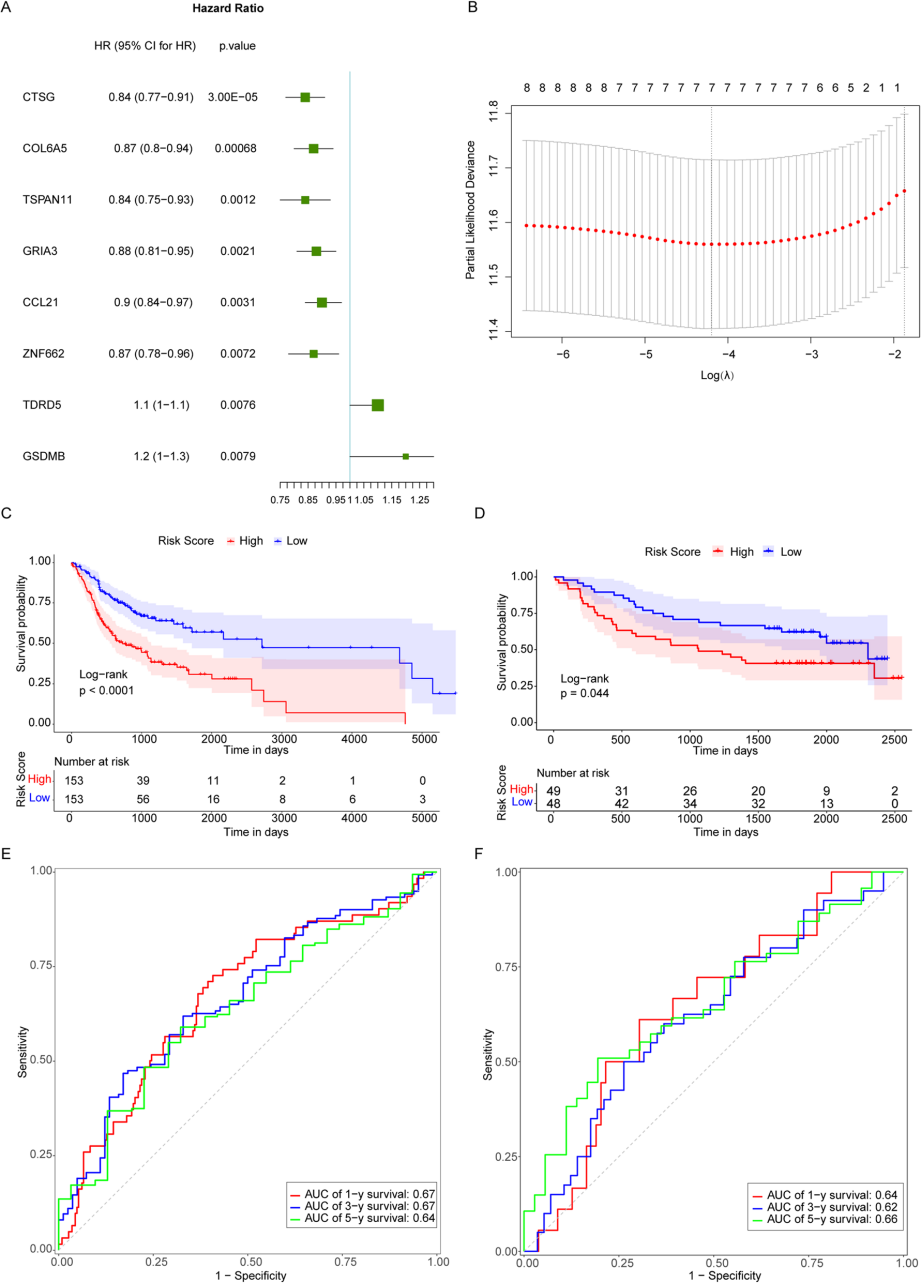
Construction of the prognostic model for OSCC. **A** Univariate analysis forest plot of 8 genes significantly associated with prognosis of OSCC. **B** A graph of LASSO regression model to determining the tuning parameter lambda. The horizontal axis and the vertical axis are log (lambda) and partial likelihood Deviance. The lambda value corresponding to the minimum value is the best which means the best Lambda value after Log is taken below the dotted line and the number of variables is corresponding to the upper part. **C** Kaplan–Meier survival curve in TCGA dataset. The horizontal axis and the vertical axis are time and survival rates. Different color represents different groups. *P* value is based on the log-rank test. **D** Kaplan–Meier survival curve in GEO dataset. The horizontal axis and the vertical axis are time and survival rates. Different color represents different groups. *P* value is based on the log-rank test. **E** Time-dependent ROC curve in TCGA dataset. The horizontal axis is specificity (rate of false alarm; 1-Specificity) and the vertical axis is sensitivity. The area under the ROC curve (AUC) value is used to assess the accuracy of prediction

Univariate Cox analysis was applied to the 306 OSCC patients in TCGA using the gene expression values of the 324 TMB-related genes as continuous variables, with *P* value < 0.01 as the threshold. Hazard ratio (HR) of each gene was calculated. The results showed as follows: CTSG (HR = 0.84, 95% CI: 0.77–0.91, *P* = 0.00003), COL6A5 (HR = 0.87, 95% CI: 0.8–0.94, *P* = 0.00068), TSPAN11 (HR = 0.84, 95% CI: 0.75–0.93, *P* = 0.0012), GRIA3 (HR = 0.88, 95% CI: 0.81–0.95, *P* = 0.0021), CCL21 (HR = 0.9, 95% CI: 0.84–0.97, *P* = 0.0031), ZNF662 (HR = 0.87, 95% CI: 0.78–0.96, *P* = 0.0072), TDRD5 (HR = 1.1, 95% CI: 1–1.1, *P* = 0.0076), GSDMB (HR = 1.2, 95% CI: 1–1.3, *P* = 0.0079) (Fig. 3A).

Then LASSO Cox regression analysis was performed on the 8 TMB-related DEGs in the training set. Based on the lambda values corresponding to the number of different genes in LASSO Cox regression analysis, the optimal number of genes was determined to 7 genes (Fig. 3B, the lowest lambda value), and the selected set of genes were CTSG, COL6A5, GRIA3, CCL21, ZNF662, TDRD5 and GSDMB.

The gene expression values were weighted with the regression coefficients to construct a prognostic Risk Score model using the following formula, Riskscore = (0.06885258) * Express Value of GSDMB + (-0.11112627) * Express Value of CTSG + (-0.05027612) * Express Value of GRIA3 + (-0.01323639) * Express Value of CCL21 + (0.03488614) * Express Value of TDRD5 + (-0.05750775) * Express Value of ZNF662 + (-0.01785164) * Express Value of COL6A5. Patients in the TCGA database and GEO validation sets were divided into high-risk and low-risk groups according to median of Risk Scores (-0.684511507). It was found that in TCGA database and GEO validation sets, patients in the high-risk group (patients with the Risk Score > 0.684511507) had lower OS rates than those in the low-risk group from the survival analysis (Fig. 3C, D). Other than that, from time-dependent receiver operating characteristic (ROC) analysis, the area under the curve (AUC) of 1-year, 3-year, and 5-year survivals of patients in the TCGA database were 0.67, 0.67 and 0.64 (Fig. 3E); the AUC of 1-year, 3-year, and 5-year survivals of patients in the GEO validation sets were 0.64, 0.62 and 0.66 (Fig. 3F). It indicated that the Risk Score model was a reliable prognostic indicator of OSCC patients in both datasets.

### Independence of the Risk Score as a prognostic indicator

**Fig. 4:**
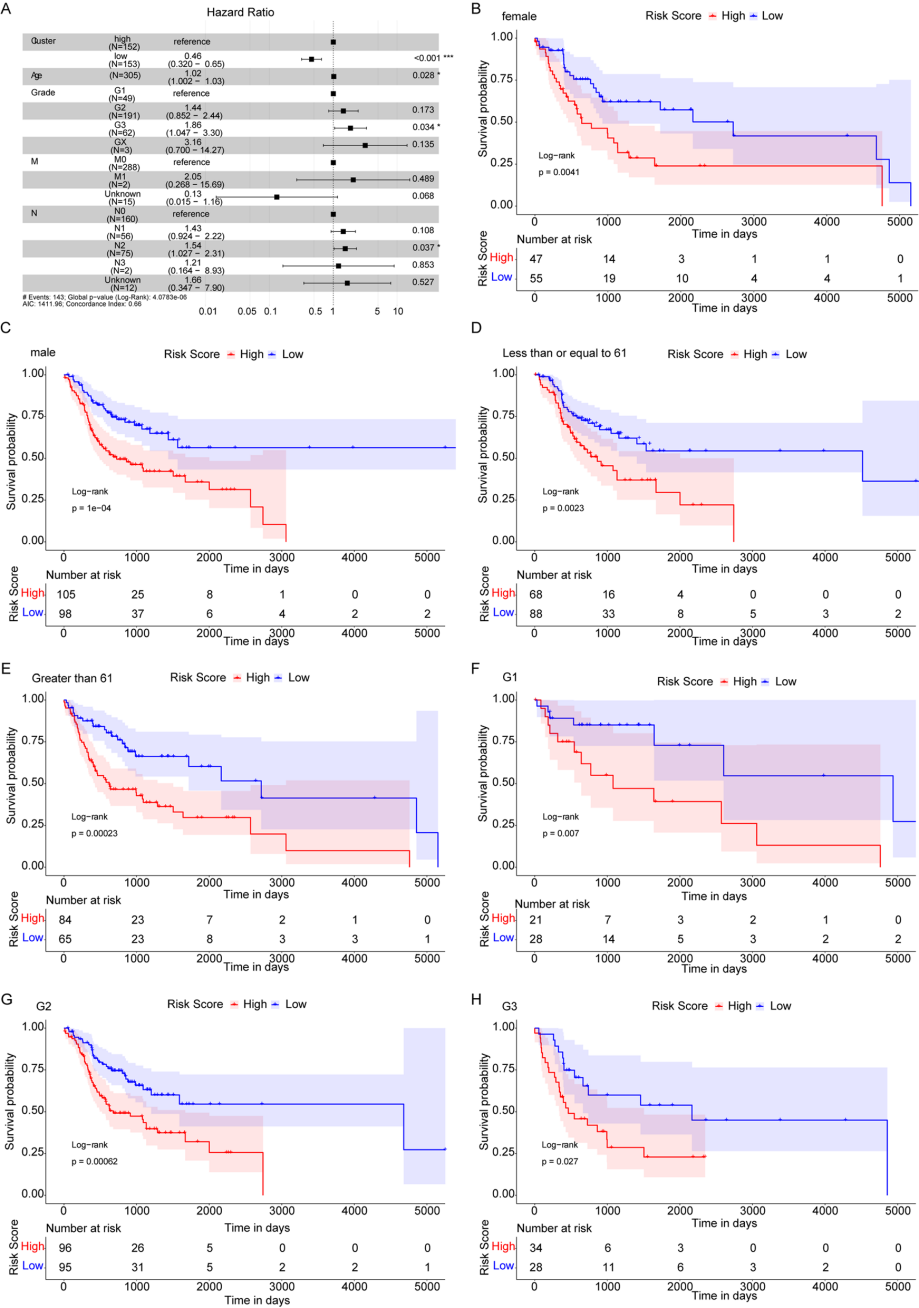
Risk Score as an independent prognostic marker for OSCC. **A** Forest plots of multivariate Cox analysis. Compared to the reference, Hazard ratio > 1 is considered to be a higher death risk while Hazard ratio < 1 is considered to be a lower death risk. **B**, **C** Kaplan–Meier survival curves of female and male subgroups. **D**, **E** Kaplan–Meier survival curves of ≤ 61 and > 61 subgroups. **F**–**H** Kaplan–Meier survival curves of G1, G2, G3 degree of differentiation subgroups

As the tumor size or/and extension (T) did not fit to the regression model, five factors comprising of age, degree of differentiation, regional lymph node involvement (N), distant metastasis (M) and median of Risk Score were included in the multivariate Cox regression analysis (one non-differentiation sample was removed) to test whether the Risk Score was an independent prognostic indicator. The results (Fig. 4A) showed that the Risk Score and degree of differentiation were significantly associated with OS. The low-risk group had lower death risk and the low Risk Score was a reliable prognostic factor (HR = 0.46, 95%CI: 0.32–0.65, *P* < 0.001). The results on fulfillment of proportional hazard’s assumption for all variables included in the multivariate Cox regression analysis were listed in Additional file 3: Table S3.

To further discuss the prognostic value of Risk Score for OSCC patients in various clinical pathological factors (including age, gender and degree of differentiation), the OSCC patients were regrouped according to age, gender and degree of differentiation to perform Kaplan–Meier survival analysis. Results showed that between female and male subgroups (Fig. 4B–C), ≤ 61 years and > 61 years old subgroups (Fig. 4D, E), as well as G1, G2 and G3 degree of differentiation subgroups (Fig. 4F–H), the OS rates of patients in the high-risk groups were all lower than those of the low-risk groups. These results indicated that Risk Score was an independent prognostic indicator for OSCC patients.

### Nomogram model predicts the prognosis of OSCC patients

**Fig. 5:**
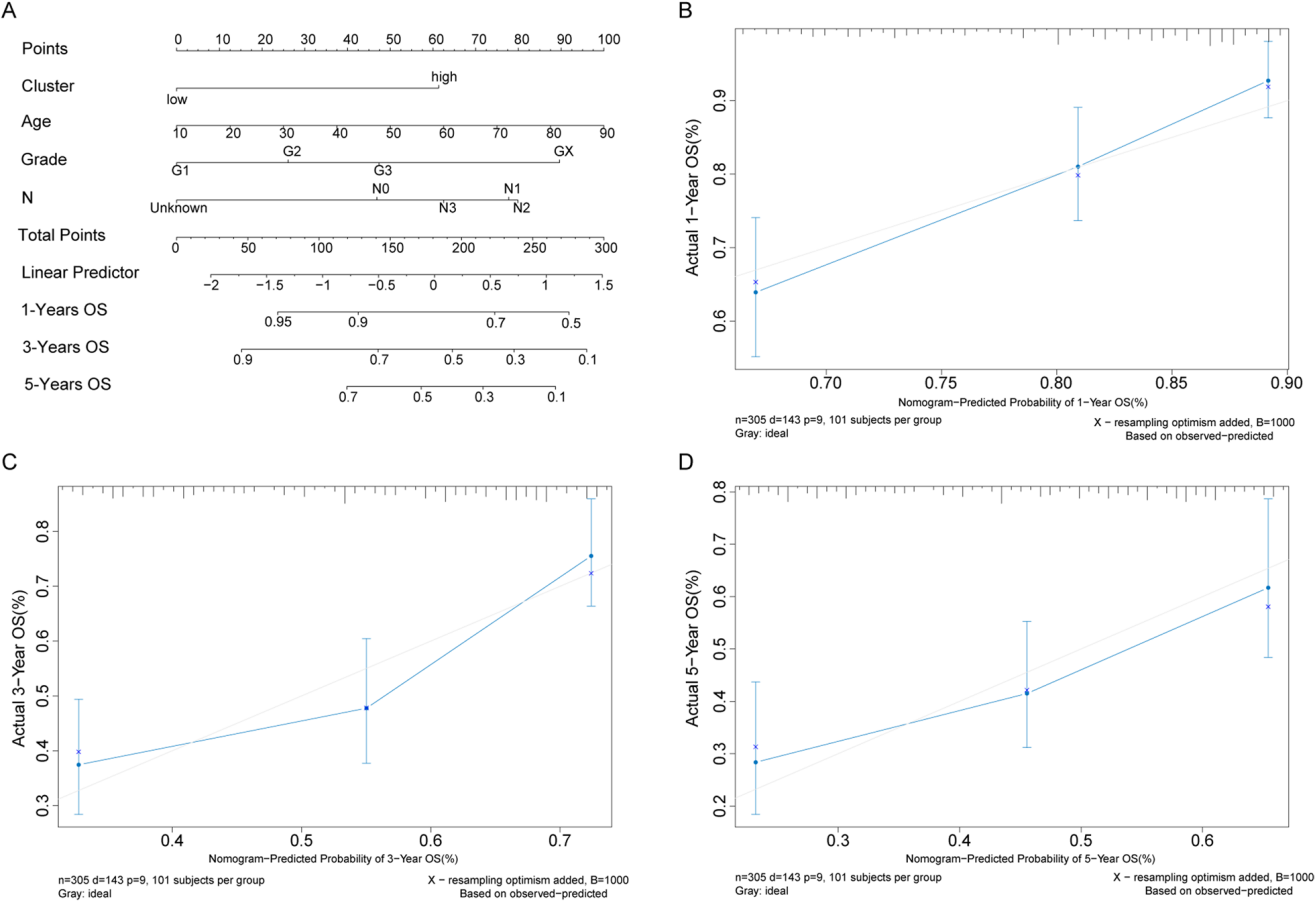
Nomogram to predict the OS of OSCC patients. **A** Nomogram to predict the OS at 1 year, 3 years and 5 years of OSCC patients. **B**–**D** The calibration plots for predicting the overall survival at 1 year, 3 years and 5 years of OSCC patients. X and y axes are the survival rates estimated by nomogram and the actual survival rates

The four independent risk factors including age, degree of differentiation, N status were used to construct nomogram model (Fig. 5A). For each patient, to obtain the actual point of Risk Score and degree of differentiation, two lines were drawn up to determine the points and the sum of the two points was located on the “Total Points” axis. Then a line was drawn down from the “Total Points” axis to the 1-year, 3-year and 5-year OS axes to predict the survival probability of OSCC patients. The 1-year and 3-year calibrated curves in adjusted diagram were close to the ideal curve (a 45 degree line through the origin of the coordinate axis with slope 1), which suggested that the predicted probabilities of the model at 1 year, 3 years and 5 years agreed well with the actual results (Fig. 5B–D).

### Immune infiltration of OSCC patients between the high- and low-risk groups

**Fig. 6:**
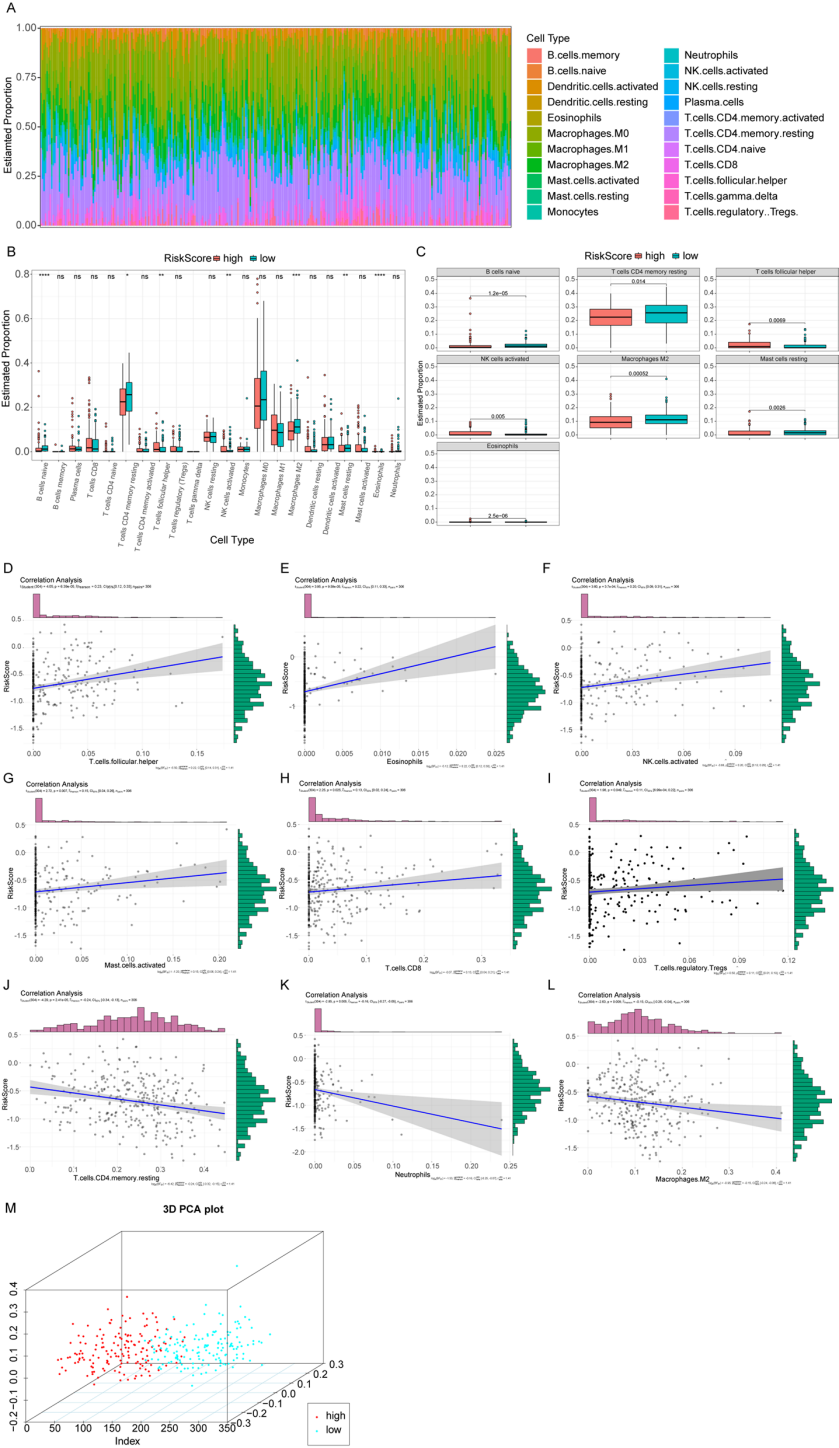
Immune infiltration between high- and low-risk groups. **A** The relative proportion of immune infiltrates in all patients. **B** Box plots of immune cell differences between high- and low-risk groups. The horizontal axis is the immune cells and the vertical axis is the relative infiltration proportion of immune cells. *P* value was calculated by wilcoxon method. (*P* > 0.05, *: *P* ≤ 0.05, **: *P* ≤ 0.01, ***: *P* ≤ 0.001, ****: *P* ≤ 0.0001). **C** Box plots of significantly different immune cells in the high- and low-risk groups. The horizontal axis and the vertical axis are the groups and the relative infiltration proportion of immune cells. *P* value was calculated by wilcoxon method. **D**–**L** The correlation diagrams of 9 immune cell and Risk Score. P value was calculated by t test and r value was calculated by the pearson correlation coefficient. **M** PCA three-dimensional clustering diagram, points of different colors represent different types of samples

CIBERSORT algorithms was used, in combination with LM22 eigenmatrix, to estimate the differences of immune infiltration of the 22 types of immune cells in high- and low-risk OSCC groups. After summarizing immune cell infiltration results of 306 OSCC patients (Fig. 6A), the variations of the proportion of tumor infiltrating immune cells in different patients may present the intrinsic characteristics of each individual. Infiltrating proportions of immune cells differed in the high- and low-risk groups (Fig. 6B). There were significant differences in the infiltrating proportions of 7 immune cells, such as B cells naive, NK cells activated and so on (Fig. 6C). The infiltration proportions of the native B cells (p = 1.2e^−05^), M2 macrophage (p = 0.00052), resting mast cells (p = 0.0026) and CD4 memory resting T cells (p = 0.014) were higher in low-risk group compared to the high-risk group, while the infiltration proportions of eosinophils (p = 2.5e^−06^), activated NK cells (p = 0.005) and follicular helper T cells (p = 0.0069) were lower in low-risk group compared to the high-risk group. Eosinophils are granulocytic cells which are connected with tumour regulation and tumor progression via inflammatory symptoms caused by degranulation [40], and increased count of eosinophils was observed in invasive OSCC in comparison with the noninvasive one [41]. The Risk Score had positive correlation with follicular helper T cell (r = 0.23, p = 6.39e^−05^), eosinophils (r = 0.22, p = 9.59e^−05^), activated NK cells (r = 0.20, p = 3.7e^−04^), activated mast cells (r = 0.15, p = 0.007), CD8 T cells (r = 0.13, p = 0.025) and regulatory T cells (r = 0.11, p = 0.049) (Fig. 6D–I). There were negative correlation between the Risk Score and CD4 memory resting T cells (r =  − 0.24, p = 2.4e^−05^), neutrophils (r =  − 0.16, p = 0.005), M2 macrophages (r =  − 0.15, p = 0.009) (Fig. 6J–L). By clustering according to the 7 immune cells which were significantly expressed, patients could be divided into two categories by principal component analysis (PCA) (Fig. 6M).

**Fig. 7:**
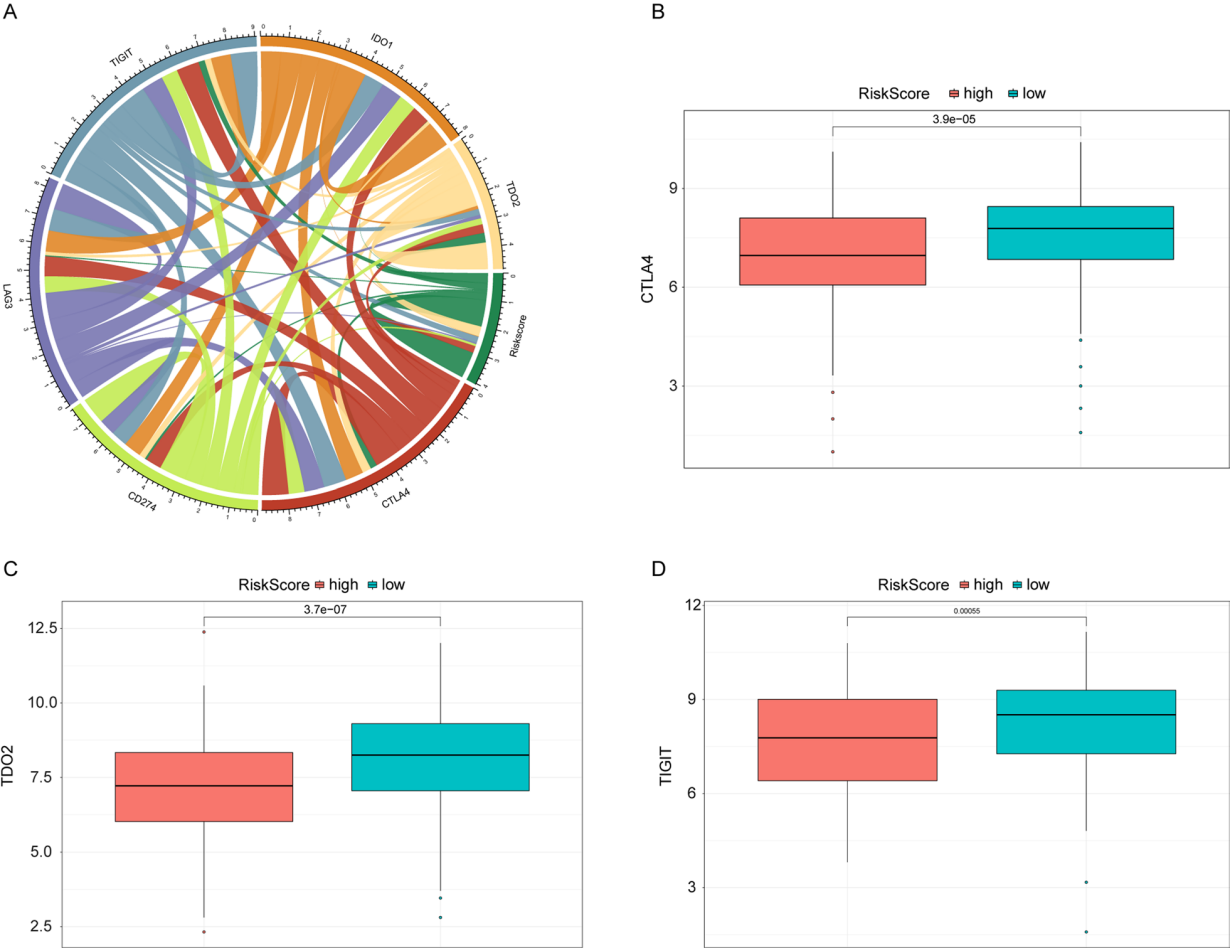
Correlation between several important immune checkpoint and Risk Score. **A** Correlation chord diagram of Risk Score and five immune checkpoints. **B**–**D** Box plots of CTLA4, TDO2 and TIGIT. Red and blue represent the high- and low-risk groups. The vertical axis is the expression of immune points. *P* value was calculated by wilcoxon method

Immune checkpoint expression has become a biomarker for selective immunotherapy in OSCC patients. The correlation between Risk Score of OSCC patients and key immune checkpoints (CTLA4, PDL1, LAG3, TIGIT IDO1 and TDO2) was analyzed and Risk Score was found to be associated with all of them (Fig. 7A). Meanwhile the expression of CTLA4, TIGIT and TDO2 differed significantly between the high- and low-risk groups (Fig. 7B–D) and they were higher in the low-risk group than in the high-risk group. CTLA4 was used in immune-related genes prognostic models for OSCC patients [14, 42].

## Discussion

OSCC is one of the most common cancers in the world. It poses a great challenge to the medical industry because of the high death rate,. In this research, we constructed a prognostic model for OSCC patients based on the TMB-related genes to predict the prognosis.

In this study, functional enrichment analysis was performed on the TMB-related DEGs. The focal adhesion was listed on the top 20 KEGG pathways. Focal adhesion kinase (FAK) is a non-receptor tyrosine kinase which is associated with poor prognosis and can promotes breast cancer cell migration and metastasis [43, 44]. Over-expression and phosphorylation of FAK also correlate with invasion and metastasis therefore affect the prognosis [45, 46]. FAK-mediated signaling and functions are involved in the development and progress of tumor [47]. Applying of the FAK inhibitor can effectively reduce the invasion and metastasis of tumor tissue [48]. These are in keeping with the our results, which indicating prognosis of OSCC is associated with the TMB-related DEGs we screened.

Seven TMB-related genes (CTSG, COL6A5, GRIA3, CCL21, ZNF662, TDRD5 and GSDMB) were selected via differential analysis, univariate Cox analysis and LASSO Cox analysis. Among the 7 genes, CTSG is confirmed as a potential biomarker in OSCC and NSCLC, and expression of CTSG is highest in adenocarcinoma [49, 50]. The expression of CCL21 is related to the poor clinical outcomes in OSCC patients via CCL21/CCR7 axis by activating the JAK2/STAT3 signaling pathway [51, 52]. ZNF662 gene caused by epigenetic changes through DNA methylation is also related to the progression of OSCC [53]. Moreover, a risk signature constructed by using COL6A5 performed well in stratifying OSCC patients with different prognosis and could distinguish survival status of OSCC patients [54]. GRIA3, as glutamate receptor, is involved in the process of tumor progression in pancreatic cancer [55]. The TDRD5 is involved in the DNA methylation and has prognostic value for patients with hepatocellular carcinoma [56]. The GSDMB is highly expressed in cancer tissues and is connected with poor prognosis by relapse-free survival, and therefore has been used as a potential novel prognostic marker [57]. These indicate that the TMB-related genes we screened may relate to the prognosis of cancer patients. The results agree with the researches that TMB-related genes have been identified in many types of cancers to help us understand progression of cancers and may assist clinical doctors to predict the prognosis of many types of cancers [58–60], which is in accord with our results.

Patients were then assigned to high- and low-risk groups according to the median of Risk Score. The results showed that patients in the high-risk group had lower OS than those in the low-risk group. The Risk Score model might be a reliable prognostic indicator for the OSCC patients. Even when taking into account other clinical variables, the Risk Score model had independent prognostic value. Head and neck squamous cell carcinoma patients with high TMB level have worse prognosis than those with low TMB [34]. And TMB-related genes have been described as a powerful prognostic biomarker for patients with bladder cancer [61]. TMB-related genes may also serve as a potential biomarker with clinical benefit in patients with NSCLC [62]. There are also many prognostic model characterizing TMB-related genes expression levels in other cancer like hepatocellular carcinoma, osteosarcoma and colon cancer [63–65]. Our results are consistent with these previous researches. It indicates that the Risk Score model constructed by 7 TMB-related genes may be helpful for the prediction of OSCC with different prognosis. Previous study has identified a 13-gene signature to predict survival of patients with OSCC [66], and the model in our study was simplified to 7 TMB-related genes. Nomogram model, built by using degree of differentiation and Risk Score, was also able to be reliable in predicting the OS of OSCC patients at 1 year, 3 years and 5 years, which makes the prognostic value of Risk Score model more reliable.

The immune cell infiltration results showed that the infiltration proportions of the native B cells, M2 macrophage, resting mast cells and CD4 memory resting T cells were higher in low-risk group compared to the high-risk group, while the infiltration proportions of eosinophils, activated NK cells and follicular helper T cells were lower in low-risk group compared to the high-risk group. The M2 macrophage is reported to promote cancer progress and to be connected with poor outcome in certain cancer types [67]. The activated NK cells may serve as anti-tumor therapy by secreting IFN-γ and TNF-α to suppress tumor cell cycle [68]. These articles are in line with our results, suggesting the Risk Score model was reliable to stratify patients by survival time.

However, there are some limitations of our study. Firstly, the main sources of our data were from public database and it was driven by statistics of retrospective data, so the best cutoff value is needed to be determined before clinical application. Secondly, the establishment and verification of the signature were based on the TCGA and GEO datasets. And the HPV status of the TCGA cohort was unknown, which makes the prognosis of the OSCC patients less reliable because the HPV status is an important risk factor affecting the prognosis of patients with head and neck cancer [69]. Thirdly, the inhomogeneity of data in the public databases also makes the Risk Score model less reliable regarding to the prognosis of cancer patients. Therefore, robustness of the signature will be necessary to be verified using larger external datasets in the future.

In conclusion, the prognostic signature reliably predicted the survival of patients with OSCC. Potential clinical use of the signature are driven by its strong prognostic performance, but the performance of the signature is still required to be verified in larger clinical samples.

## Conclusion

Our study suggests that the 7 TMB-related genes are associated with the prognosis of OSCC and Risk Score model constructed using TMB-related genes (CTSG, COL6A5, GRIA3, CCL21, ZNF662, TDRD5 and GSDMB) might be a reliable biomarker for predicting the prognosis of OSCC patients. The prognostic signature may be helpful in designing the individualized treatment management and in making of medical decisions in the future but the implement of prognostic stratification of OSCC patients is still required to be verified in more clinical settings.

## Supplementary Information

**Additional file 1: Table S1**. The full results of GO enrichment analysis.

**Additional file 2: Table S2**. The full results of KEGG enrichment analysis.

**Additional file 3: Table S3**. The fulfillment of proportional hazard’s assumption for all variables in multivariate Cox regression analysis.

## Data Availability

The datasets analyzed during the current study are available in the Cancer Genome Atlas (TCGA, https://tcga-data.nci.nih.gov/tcga/) and the Gene Expression Omnibus (GEO, http://www.ncbi.nlm.nih.gov/geo/).
